# Differential Involvement of EEG Oscillatory Components in Sameness versus Spatial-Relation Visual Reasoning Tasks

**DOI:** 10.1523/ENEURO.0267-20.2020

**Published:** 2021-01-21

**Authors:** Andrea Alamia, Canhuang Luo, Matthew Ricci, Junkyung Kim, Thomas Serre, Rufin VanRullen

**Affiliations:** 1 CerCo, Centre National de la Recherche Scientifique Université de Toulouse, Toulouse 31055, France; 2 Department of Cognitive, Linguistic and Psychological Sciences, Carney Institute for Brain Science, Brown University, Providence, RI 02912; 3 Artificial and Natural Intelligence Toulouse Institute (ANITI), Université de Toulouse, Toulouse 31055, France

**Keywords:** deep neural networks, EEG oscillations, ERPs, spatial relationship, visual reasoning

## Abstract

The development of deep convolutional neural networks (CNNs) has recently led to great successes in computer vision, and CNNs have become de facto computational models of vision. However, a growing body of work suggests that they exhibit critical limitations on tasks beyond image categorization. Here, we study one such fundamental limitation, concerning the judgment of whether two simultaneously presented items are the same or different (SD) compared with a baseline assessment of their spatial relationship (SR). In both human subjects and artificial neural networks, we test the prediction that SD tasks recruit additional cortical mechanisms which underlie critical aspects of visual cognition that are not explained by current computational models. We thus recorded electroencephalography (EEG) signals from human participants engaged in the same tasks as the computational models. Importantly, in humans the two tasks were matched in terms of difficulty by an adaptive psychometric procedure; yet, on top of a modulation of evoked potentials (EPs), our results revealed higher activity in the low β (16–24 Hz) band in the SD compared with the SR conditions. We surmise that these oscillations reflect the crucial involvement of additional mechanisms, such as working memory and attention, which are missing in current feed-forward CNNs.

## Significance Statement

Convolutional neural networks (CNNs) are currently the best computational models of primate vision. Here, we independently confirm prior results suggesting that CNNs can learn to solve visual reasoning problems involving spatial relations much more easily than problems involving sameness judgments. We hypothesize that these results reflect different computational demands between the two tasks and conducted a human electroencephalography (EEG) experiment to test this hypothesis. Our results suggest a significant difference, both in evoked potentials (EPs) and in the oscillatory dynamics, of the EEG signals measured from human participants performing these two tasks. We interpret this difference as the signature for the fundamental involvement of recurrent mechanisms implementing cognitive functions such as working memory and attention.

## Introduction

The field of artificial vision witnessed an impressive boost in the last few years, driven by the striking results of deep convolutional neural networks (CNNs). Such hierarchical neural networks process information sequentially, through a feedforward cascade of filtering, rectification, and normalization operations. The accuracy of these architectures is now approaching, sometimes exceeding, that of human observers on key visual recognition tasks including object (He et al., 2016) and face recognition (Phillips et al., 2018). These advances suggest that purely feedforward mechanisms suffice to accomplish remarkable results in object categorization, in line with previous experimental studies on humans (VanRullen and Thorpe, 2001) and animals (Hollard and Delius, 1982; Vogels, 1999). However, despite the remarkable accuracy reached in these recognition tasks, the limitations of CNNs are becoming increasingly evident (for recent review, see Serre, 2019). Beyond image categorization tasks, CNNs appear to struggle to learn to solve relatively simple visual reasoning tasks otherwise trivial for the human brain (Stabinger et al., 2016; Kim et al., 2018). A recent study (Kim et al., 2018) thoroughly investigated the ability of CNN architectures to learn to solve various visual reasoning tasks, and found an apparent dichotomy between two sorts of problems: on the one hand, tasks that require judging the spatial relations between items [spatial relationship (SR)]; on the other, those that require comparing items [same-different (SD)]. Importantly, Kim and colleagues demonstrated that CNNs can more easily learn the first class of problems compared with the second one.

This prompts the question of how biological visual systems handle such tasks so efficiently. Kim et al. (2018) suggest that SR and SD tasks tap into distinct computational mechanisms, thus leading to the prediction that different cortical processes are also involved when humans perform the two tasks: SR tasks can be successfully solved by feedforward processes, whereas SD tasks seem to require additional computations, such as working memory and attention. Here, we tested this hypothesis in two steps: first, we confirmed and extended Kim’s results by comparing the performance of CNNs on an experiment in which we directly contrasted SD and SR tasks on the same stimulus set. Second, we recorded electrophysiological responses [electroencephalography (EEG)] in healthy human participants for the same experiment, after having matched the difficulty level via an adaptive psychometric procedure. We hypothesized that the additional computations required by the SD task, as compared with SR tasks, would elicit differences in evoked potentials (EPs; e.g., P300 modulations, which have been related to attentional mechanisms; Nash and Fernandez, 1996) and brain rhythms related to working memory (such as β band oscillations; Benchenane et al., 2011; Lundqvist et al., 2018). We found indeed that, in addition to a variation in EPs, the SD task elicited higher activity in specific β band oscillatory components in the occipital-parietal areas, which are typically associated with attention-related and memory-related processes. We emphasize that the goal of the present study was not to identify the precise neural computations involved in the two tasks (which would naturally require a broader experimental set-up than a single EEG study), but rather to validate the hypothesis that SD involves additional computations relative to SR (even when the two tasks are equally difficult). We hope that this demonstration can be a first step toward characterizing the processes taking place in visual cortex during visual reasoning tasks, and designing more reliable and more human-like computational models.

## Materials and Methods

### Participants and pilot experiment

Twenty-eight participants (aged 21–34 years old with a mean age of 26.6 ± 3.7, 11 women, five left-handed), volunteered to join the experiment. All subjects reported normal or corrected to normal vision and had no history of epileptic seizures or neurologic disorders. Participants were pooled in two groups of 14 each: one group performed a pilot experiment, while the second one was tested on a final version of the task. The only difference between the pilot and the main study was the QUEST adaptive procedure used to match the difficulty level between conditions, which was not implemented in the pilot experiment. However, in both studies we found the very same result (see below, Figs. 3, 4 and 5). In the main experiment, we kept the same number of participants to replicate the effect, after having removed the behavioral difference in task difficulty via the QUEST algorithm. This study complies with the guidelines of the research center where it was conducted, and the protocol was approved by an external committee (ethics approval number N° 2016-A01937-44). All participants gave written informed consent before starting the experiment, in accordance with the Declaration of Helsinki, and received monetary compensation for their participation.

### Experimental design

**Figure 1.**
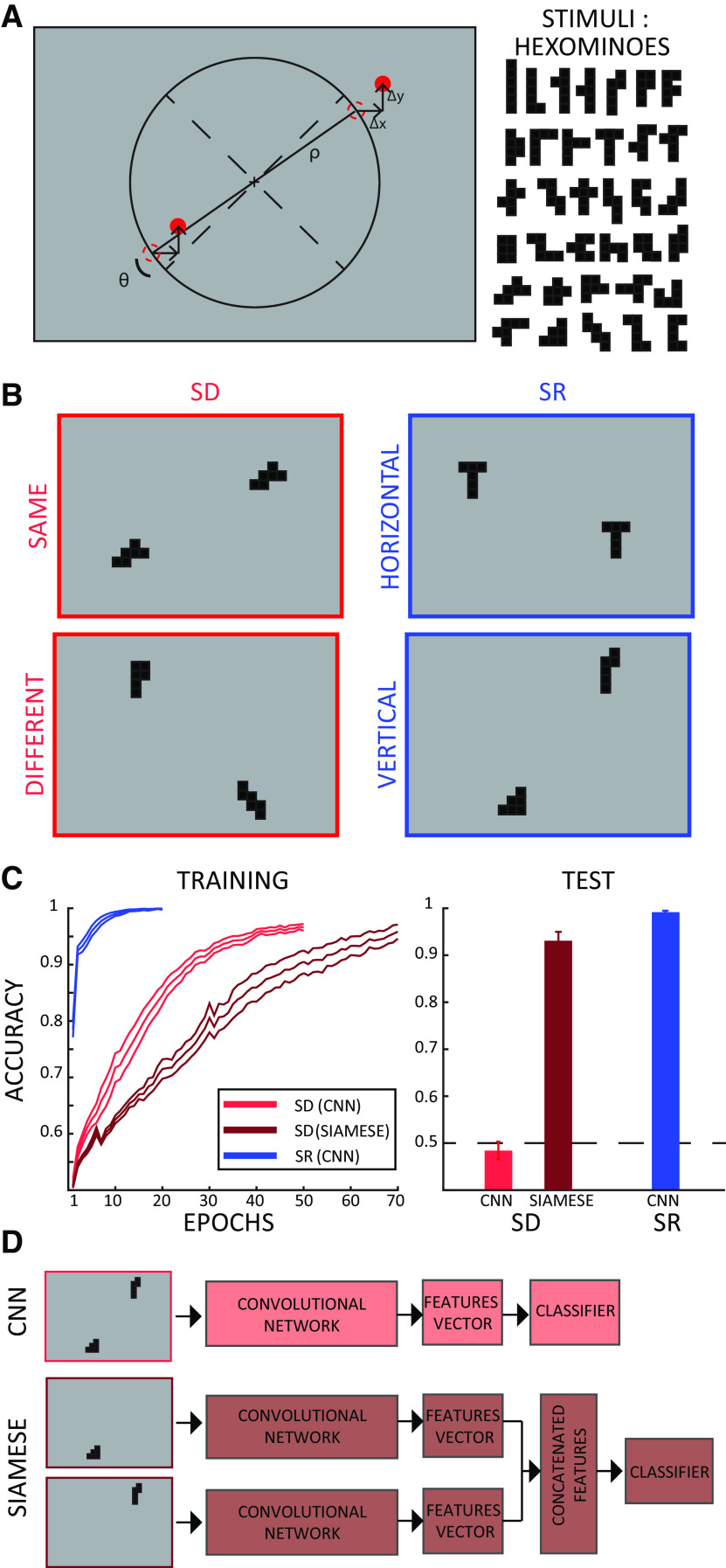
Stimuli and simulation results. ***A***, The stimuli were the same in the simulations and in the human experiments. The items were displayed at opposite sides of the screen (either 45° and 225° or −45° and −225°). Both item positions were jittered by a random amount in both the *x*- and *y*-axes (Δx and Δy in the picture) to make the task non-trivial for human participants (i.e., preventing participants from performing the SR task considering only the position of one item, thus ignoring the SR between the two items). The items used are hexominoes (right panel). Minimum and maximum item height and width are 1.2–3.6° and 1.2–2.7° of visual angle, respectively, and 2–5 pixels used for the simulations (image size was 50 × 80 pixels). ***B***, Example of stimuli position for the SD task (left column) and spatial relation task (SR, right column). For the sake of illustration, the ratio between the screen and hexominoes size has been modified (stimuli here look bigger than in the real experiment). ***C***, ***D***, Accuracy of the CNN network on the SD (light red) and SR (blue) tasks, and of a Siamese network trained on the SD task (dark red). The Siamese network mimics segmentation in a feedforward network, by separating the items in two distinct channels of the network (see ***D***). The left panel shows the training curves for each network (accuracy over epochs during training); we stopped the training when the validation accuracy reached 90%. In the right panel, we show the training accuracy at the last epoch and the test accuracy. The latter was evaluated using novel items never used for training, and it reveals that the CNN seems to only learn the required rule for the SR but not for the SD task, as shown in a previous study. Conversely, the Siamese network (CNN with segmentation) can solve the SD task, demonstrating that segmentation can allow the CNN to successfully accomplish this task. In both panels we show average values ± SE over 10 repetitions using different random initializations.

The experiment was composed of 16 experimental blocks of 70 trials each, with a total duration of ∼1 h. Each trial lasted ∼2 s (Fig. 1*A*): 350 ms after the onset of a black fixation cross (0.6° width), two shapes were displayed for 30 ms on opposite sides of the screen, distant 2*ρ from each other with an angle of ±(45° + θ) with respect to the horizontal midline (ρ being the distance from the center of the screen, and θ the angular difference with the diagonal; see Fig. 1*B*). Each shape was selected from a subset of 36 hexominoes, a geometric figure composed of six contiguous squares (Fig. 1*B*) One second after the onset of the hexominoes, the fixation cross turned blue, cuing participants to respond. In half of the blocks, participants had to report whether the two shapes were the same or different (SD condition); in the remaining blocks participants had to judge whether the two stimuli were aligned more horizontally or vertically (SR condition). Shapes were displayed at opposite sides of the screen along two main possible orientation axes sampled at random for every trial (either 45° and 225° or −45° and −225°). Both stimuli positions were jittered by a random offset Δx and Δy in both the x and *y*-axis and a rotation θ from the main axis. The same offsets were applied to both shapes, so they did not affect the angle between stimuli. The aim of such offsets was to prevent participants in the SR condition from determining the configuration of the two stimuli (orientation task) by merely judging the position of a single stimulus: without the random offsets, considering for example the top-right corner position, if the item were below/above the (imaginary) screen diagonal line, the overall orientation would be horizontal/vertical, without the need to consider the position of the corresponding bottom-left item. The offset then compelled participants to consider the relative position of both hexominoes at once. Importantly, in the main experiment (compared with the pilot experiment) the difficulty of the two tasks was controlled by an adaptive psychometric procedure (QUEST method; Watson and Pelli, 1983), which varied the eccentricity of the two stimuli ρ (in the SD blocks) or θ (in the SR blocks) to maintain an overall accuracy level of 80% throughout the whole experiment. In fact, larger (smaller) values of ρ made the stimuli more (less) eccentric and the task more (less) difficult; similarly, smaller (larger) values of θ set the stimuli closer to (farther from) the 45° diagonal line, making the task more (less) difficult. We modified one parameter per condition (i.e., per block), while the other was kept constant (using the same value as in the preceding block). After participants responded, they received feedback on their performance: the fixation cross turned green (red) in case of a correct (incorrect) answer. Throughout the experiment the condition blocks were alternated, the first block being the SD condition for all participants. Before starting the first block, participants performed one training block per condition. The purpose of this training was (1) to familiarize participants with the experimental conditions, (2) to initialize the ρ and θ parameters in the QUEST method for the first experimental block (initial values were respectively ρ = 5.4° of visual angle and θ = 6° of rotation). All experiments were performed on a cathode ray monitor, positioned 57 cm from the subject, with a refresh rate of 160 Hz and a resolution of 1280 × 1024 pixels. The experiment was coded in MATLAB using the Psychophysics Toolbox (Brainard, 1997). The stimuli were presented in black on a gray background. Throughout the experiment we recorded EEG signals.

### EEG recording and preprocessing

We recorded brain activity using a 64-channel active BioSemi EEG system (1024-Hz digitizing rate, three additional ocular electrodes). The preprocessing was performed in MATLAB using the EEGlab toolbox (Delorme and Makeig, 2004). First, the data were downsampled to 256 Hz. A notch filter (47–53 Hz) was then applied to remove power line artifacts. We applied an average-referencing and removed slow drifts by applying a high-pass filter (>1 Hz). We created the data epochs aligning the data to the onset of the fixation cross. Finally, we performed an ICA decomposition to remove components related to eye movements and blink artifacts: we visually inspected the data and removed from two to five components per subject with a conservative approach (we removed only components in the frontal regions clearly related to eye movements’ activity).

### Computational modeling and code accessibility

We extended a previous computational study (Kim et al., 2018) from which we chose the parameters of the convolutional feedforward network trained on the SD and SR tasks. Each task was run 10 times, randomly initializing the networks’ parameters and the stimuli used in the training and test set. The network was fed with 50 × 80 pixel images. Two hexominoes (width and height of two to five pixels) were placed at opposite sides of the screen (Fig. 1*A*; see above, Experimental design). The dictionary of hexominoes was composed of 35 items, which were randomly split between a training (30 items) and a test set (five items) at each iteration. Both the training, validation and test sets were composed of 1000 stimuli (i.e., different combinations of the hexominoes, with slightly different eccentricity and/or offset relative to the diagonal). The network consisted of six convolutional layers. Each layer contained four channels of size 2 × 2, with stride of 1. All convolutional layers used a ReLu activation function with stride of 1 and were followed by pooling layers with 2 × 2 kernels and a stride of 1. Eventually, two fully connected layers with 128 units preceded a two-dimensional classification layer with a sigmoid activation function. As a regularizer we set a dropout rate of 0.3 in each layer of the network. We used binary cross-entropy as a loss function, the adaptive moment estimation (Adam) optimizer (Kingma and Ba, 2015) and a learning rate of 10e-4. Each simulation was run over 70 epochs with batch size of 50. All simulations were run in TensorFlow (GoogleResearch, 2016). The Siamese network had the same exact convolutional architecture as described above; additionally, the difference between features-vectors of each separate item (computed on an input image where this item was shown alone) was fed to the classifier to perform the SD task. All networks count ∼7e06 parameters. All the code and data required to replicate the simulations are available at a GitHub repository (https://github.com/artipago/SD-SR). The code has been run on a Window PC on Python using the “Tensorflow,” “Keras,” “Scipy,” and “Numpy” libraries.

### Statistical analysis, behavior

We analyzed both accuracy and reaction times (RTs) by means of Bayesian ANOVA, considering the block condition (SR and SD, see above) as independent variables and the trial condition (whether the stimuli were same or different, or more horizontally or vertically aligned). The result of such analysis provides a Bayes factors (BF), which quantifies the ratio between statistical models given the data. Throughout the paper, all BFs reported correspond to the probability of the alternative hypothesis over the null hypothesis (indicated as BF_10_). Practically, a large BF (∼BF > 5) provides evidence in favor of the alternative hypothesis (the larger the BF the stronger the evidence), whereas low BF (∼BF < 0.5) suggests a lack of effect (Bernardo and Smith, 2009; Masson, 2011). We performed all Bayesian analyses in JASP (Love et al., 2015; JASP Team, 2018).

### Statistical analysis, electrophysiology

Regarding the EEG recording we performed two analyses: one in the time domain measuring evoked related potentials, ERPs, and the other one in the frequency domain using a time-frequency transform. In the first case, we considered the ERPs recorded from seven midline electrodes (i.e., Oz, POz, Pz, CPz, Cz, FCz, and Fz). After subtracting the baseline activity recorded during the 350 ms before stimuli onset, we averaged the signals from the SD and SR blocks respectively (i.e., eight blocks for each condition). Finally, we tested whether the difference between these signals differed from 0 by means of a point-by-point two-tailed *t* test with a false discovery rate (FDR) correction for multiple comparisons (Hochberg, 1995). Regarding the time-frequency analysis, we computed the power spectra by means of a wavelet transform (1–50 Hz in log-space frequency steps with 1–20 cycles). After baseline correction (i.e., dividing by the averaged activity of the 350 ms before the onset of the fixation cross), for each participant, we computed the difference in decibel of the two conditions point by point, averaging over all electrodes. As in the ERP analysis, we performed a point-by-point two-tailed *t* test to identify the time-frequency regions which were significantly different. We applied a cluster-based permutation to correct for multiple comparisons (Maris and Oostenveld, 2007). First, we identified clusters composed of *t* values *t* > 3.5 (*p* < 0.01), and for each one we computed the respective global sum. In order to estimate the null distribution over the combined *t* values, we performed the same procedure 500 times after shuffling the subject by subject SD-SR assignment. Eventually, we obtained the *p* values for each non-shuffled cluster given the null distribution. All EEG analyses were performed in MATLAB; the wavelet transform was performed using the EEGlab toolbox (Delorme and Makeig, 2004).

## Results

### Computational modeling

We first extended the results by Kim et al. (2018) for our novel stimulus set: we trained two separate CNNs architectures to solve an SD and an SR task using a single stimulus set (Materials and Methods). The input to these networks was an image (50 × 80 pixels) in which two hexominoes (width and height of two to five pixels) were displayed at opposite sides of the screen (Fig. 1*A*). The networks were trained to classify whether the two hexominoes were the same or not (SD task) or whether they were aligned more vertically or more horizontally with respect to the midline (SR task).

We trained and tested the network on different sets of items (a training and test set, respectively) to assess the networks’ ability to generalize beyond training data. We trained and tested the networks 10 times, randomly initializing networks, parameters as well as the training and test set split each time. We report the mean accuracy and standard deviation over these 10 repetitions in Figure 1*B*. Our results are consistent with those from Kim et al. (2018): a CNN appears to be able to learn the abstract rule (as measured by the network’s ability to generalize beyond the shapes used for training) for SR tasks much more easily than SD tasks. The effortless ability of humans and other animals (Wasserman et al., 2012; Daniel et al., 2015) to learn SD tasks suggest the possible involvement of additional computations that are lacking in CNNs, possibly achieving items identification or segmentation (e.g., via attention and working memory). In order to verify that segmentation could be a missing ingredient for the SD task, we implemented a variant of the CNN with built-in segmentation properties, and tested it on the SD task (it is not necessary to test it on the SR task, because generalization performance is already at ceiling). The new network used a Siamese architecture (Bromley et al., 1993) in which each item is processed separately and eventually combined before being passed to a classifier. Therefore, this model mimics the effect of selective attention and item segregation by feeding to the network each item separately. The Siamese network could achieve the same training performance on the SD task as the standard CNN (although the training took more epochs); however, the network was able to generalize to the test set, while the standard CNN test accuracy was at chance. This supports the idea that item segmentation or individuation abilities are needed to achieve the SD task Next, we test the prediction that SD tasks in humans also require additional computational mechanisms than SR tasks by recording EEG signals from a pool of 28 participants (14 of which were tested on a pilot experiment; Fig. 5) performing the same SD and SR tasks.

### Human behavior

**Figure 2.**
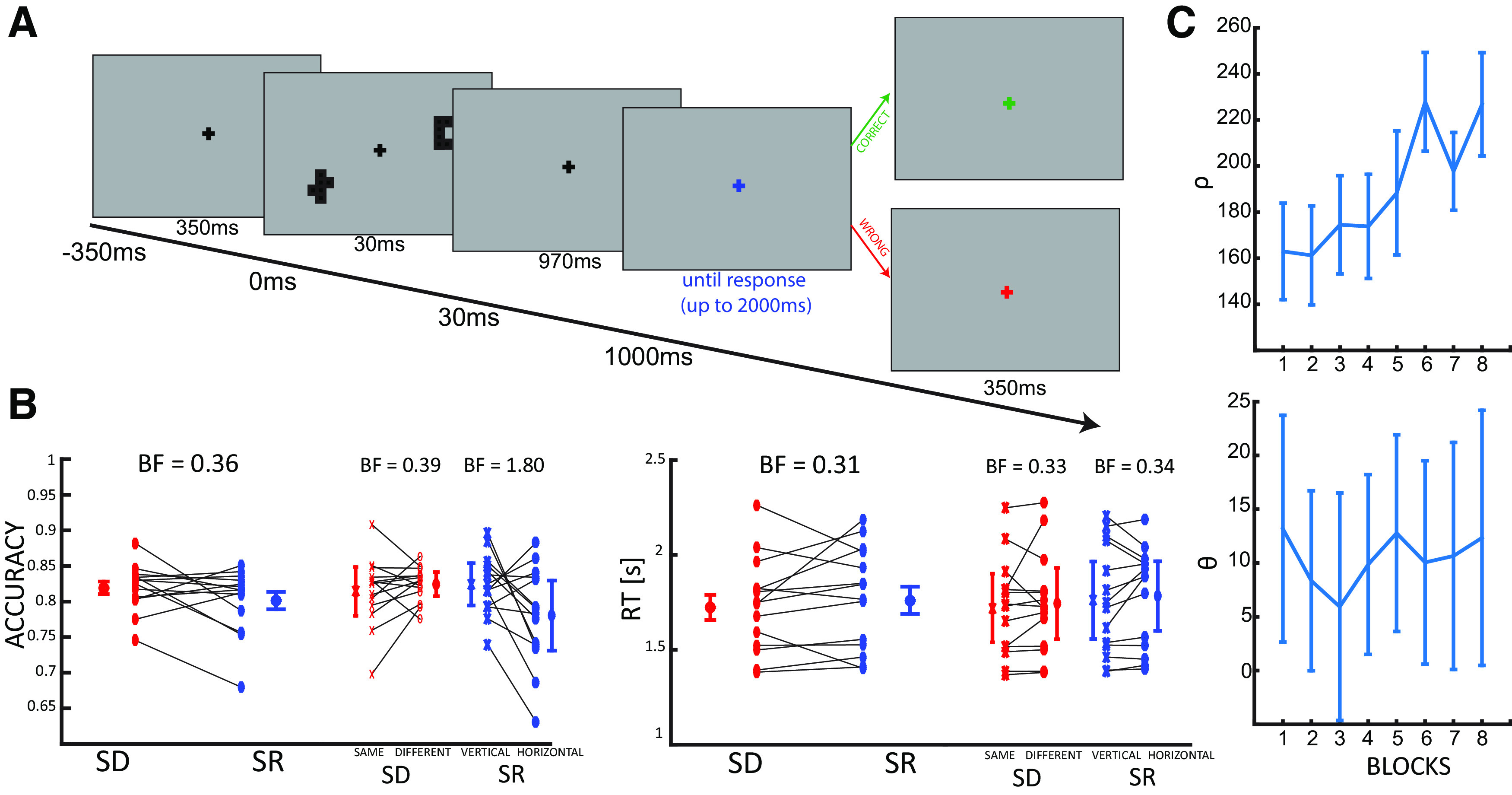
Experimental design and human behavioral results. ***A***, At the beginning of each trial, a black fixation cross was displayed for 350 ms. After two stimuli were shown for 30 ms, participants waited an additional 970 ms before providing the answer. The response was cued by the fixation cross turning blue. After the response, the color of the fixation cross provided feedback: green if the response was correct, red otherwise. ***B***, Humans performed the SD and SR tasks with comparable levels of performance. In the left and right panels are shown the averages ± SE for accuracy and RTs, respectively. Each pair of connected markers represent an individual subject. The results for the SD (in red) and SR (in blue) conditions are further broken down for each condition separately (SD and vertical-horizontal). BF indicates the BF against the null hypothesis (difference between the two conditions). ***C***, Changes over blocks of ρ (the distance between the stimuli, left panel) and θ (the angle between the stimuli and the meridian, right panel) as adjusted by the QUEST algorithm.

A first pilot group of 14 participants performed the SD and SR tasks as described in Figure 2*A*, but without any procedure for adjusting task difficulty (i.e., the QUEST method). The same EEG oscillatory differences between the two tasks as in the main experiment were observed (Fig. 5); however, concomitant differences in behavioral task performance left open the possibility that the oscillatory effects were caused by differences in task difficulty (Fig. 5*A*). Therefore, we replicated the experiment on another group of 14 subjects, this time with an adaptive procedure to equate behavioral performance between the SD and SR tasks.

Participants (*N* = 14) in this main experimental task completed 16 blocks using the same stimuli as those used to train CNNs (Fig. 1): in half of the blocks they were asked to report whether the two hexominoes were the same or not (SD conditions), in the other half whether the hexominoes were more vertically or horizontally aligned (SR conditions). The two conditions were interleaved in a block design. Participants were required to answer after one second from stimulus onset to disentangle motor from visual components in the EEG recordings (Fig. 2*A*). The QUEST algorithm was used to assure that participants’ accuracy was matched between the two tasks and remained constant throughout the whole experiment. This was done by adjusting two experimental parameters trial by trial (i.e., the hexominoes eccentricity in SD blocks, ρ, and the angle from the diagonal in SR blocks, θ; see Figs. 1*A*, 2*C*). Maintaining a comparable accuracy between the two tasks reduces the potential for confounds in the electrophysiological analysis because of differences in performance, vigilance, or motivation. We confirmed the absence of any substantial behavioral difference between the SD and SR tasks (Fig. 2*B*) with a Bayesian ANOVA on both accuracy (BF_10_ = 0.361, error < 0.001%) and RT (BF_10_ = 0.317, error < 0.89%). In addition, we also investigated each condition separately (Fig. 2*B*), comparing the difference between “same” and “different” trials (in SD blocks) and “vertical” and “horizontal” trials (in SR blocks) in both RT and accuracy. All comparisons revealed overall no differences between tasks, except for the accuracy of vertical and horizontal trials in the SR condition, in which the BF proved inconclusive (accuracy: SD, BF_10_ = 0.39, error < 0.012%; SR, BF_10_ = 1.80, error < 0.001%; RT: SD, BF_10_ = 0.333, error < 0.01%; SR, BF_10_ = 0.34, error < 0.01%).

### Human electrophysiology: EPs

**Figure 3.**
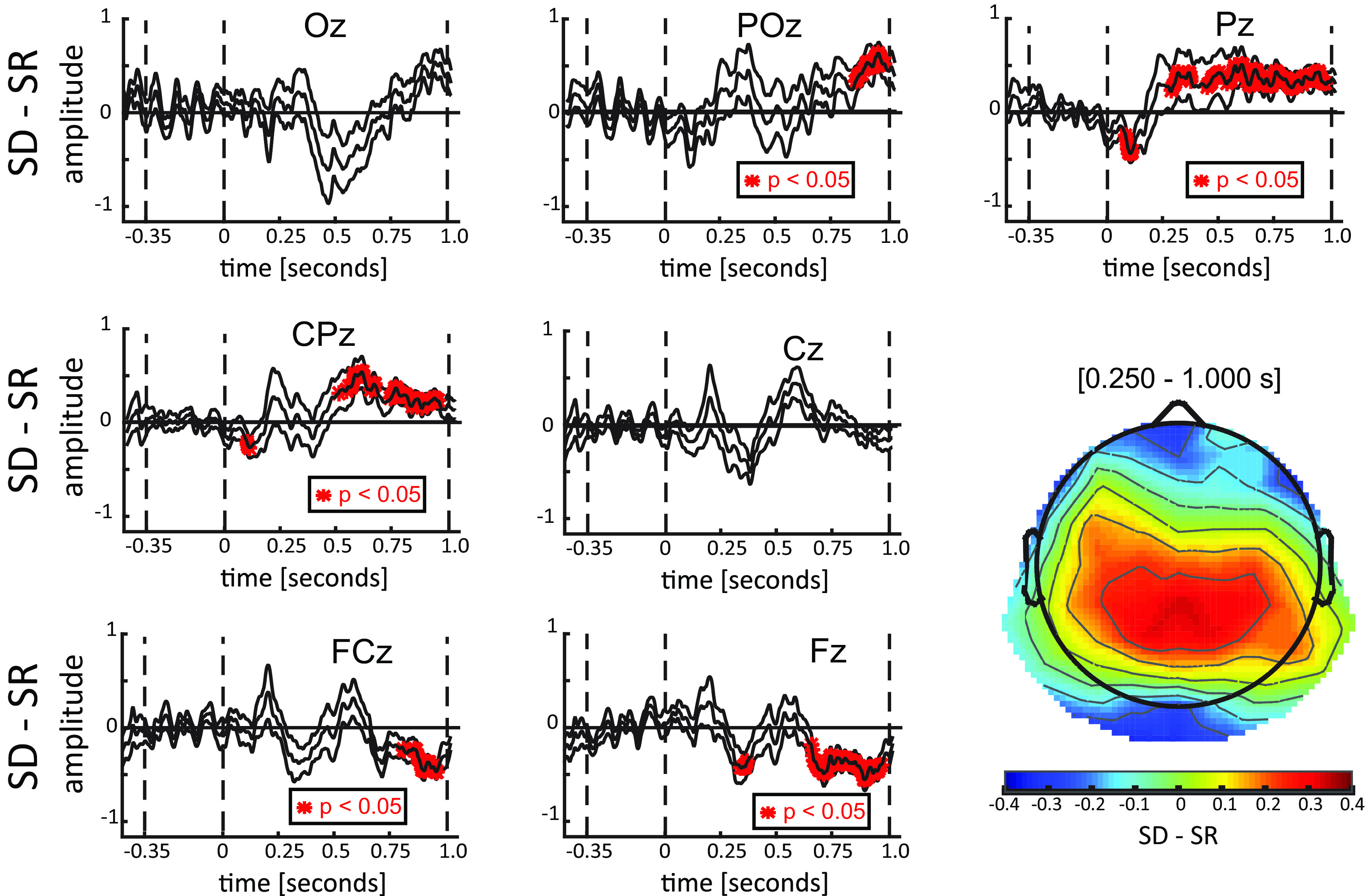
ERPs results. Each panel represents the difference between ERPs elicited in the SD and SR conditions for the seven midline electrodes (average ± SE). Shown in red are the points for which a significant difference was found against zero. The results reveal a significant difference from 250 ms after stimuli onset until the response cue (at 1000 ms) in central parietal regions, and an opposite effect after 750 ms in frontal regions. In the bottom-right panel, the topography, computed over the 250- to 1000-ms interval, confirmed a larger activity in the SD than in the SR condition (positive difference, warmer colors) in the central-parietal regions, and an opposite effect (negative difference, colder colors) in the frontal regions (which, although not significantly, also included occipital regions).

After having confirmed that performance was equal in the two tasks, we characterized the EPs in each task. First, we estimated the difference between SR and SD conditions considering 7 midline electrodes (Fig. 3). The results of a point-by-point *t* test corrected for multiple comparisons revealed a significant difference in central and posterior electrodes (mostly Pz and CPz) between 250 ms after the onset of the stimuli and the response cue, and the opposite effect in frontal electrodes (FCz and Fz) from 750 to 1000 ms, as confirmed by the topography (Fig. 3). Overall, these results indicate larger potentials in visual areas during the SD task than in the SR. Previous studies have shown a relation between EP amplitude (particularly P300 and late components) with attention (Van Voorhis and Hillyard, 1977; Krusemark et al., 2016; Itthipuripat et al., 2017, 2018) and visual working memory (Fabiani et al., 1986; McEvoy et al., 1998; Kok, 2001). Our results are thus consistent with a larger involvement of executive functions in the SD versus SR task. In the following, we investigated whether this hypothesis is corroborated by corresponding oscillatory effects in the time-frequency domain in the main experiment.

### Human electrophysiology: time-frequency analysis

**Figure 4.**
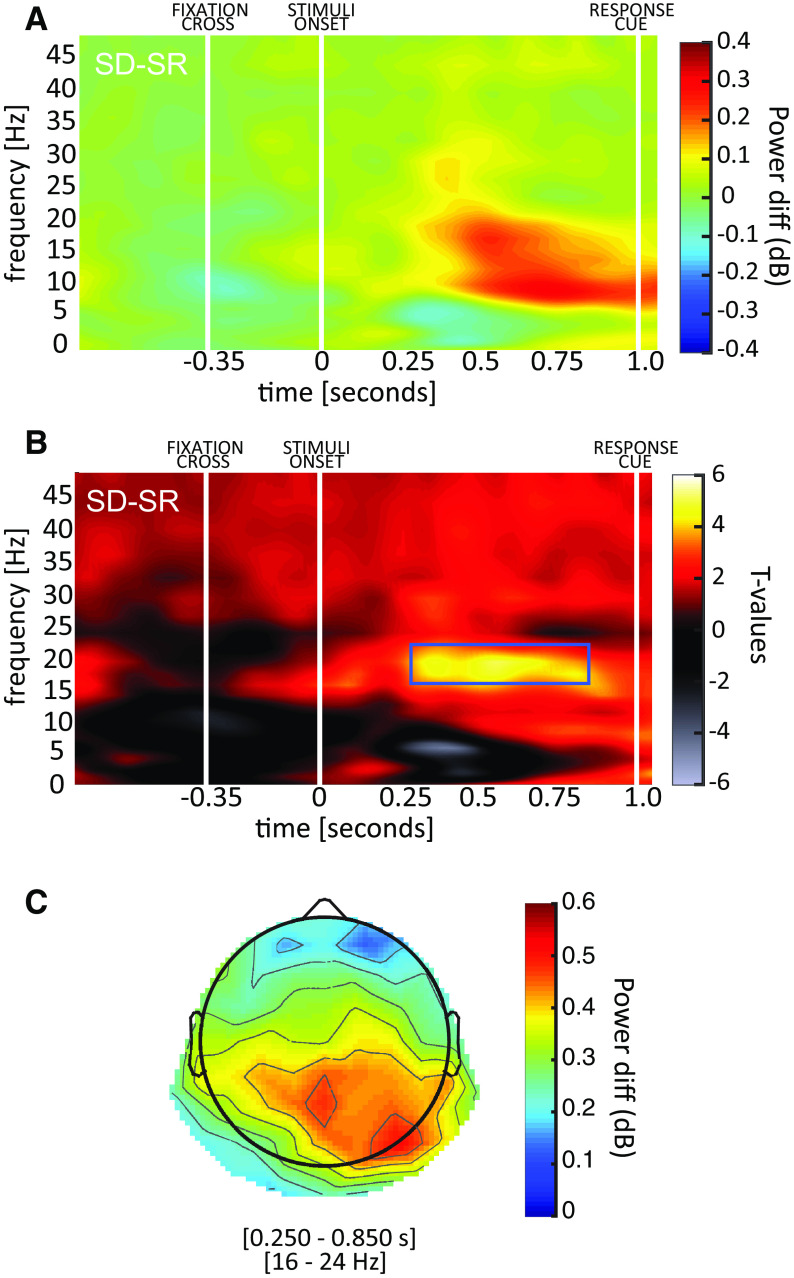
Time-frequency results. ***A***, The difference between SD and SR power spectra is shown in the first panel. White lines indicate the onset of the fixation cross, the stimuli and the response cue. ***B***, The second panel shows the corresponding *t* values (when testing the difference against zero). We observed a significant region in the low β band (16–24 Hz), between 250 and 950 ms after stimulus onset. ***C***, The topography of the significant time-frequency window reveals the involvement of occipital-parietal regions.

**Figure 5.**
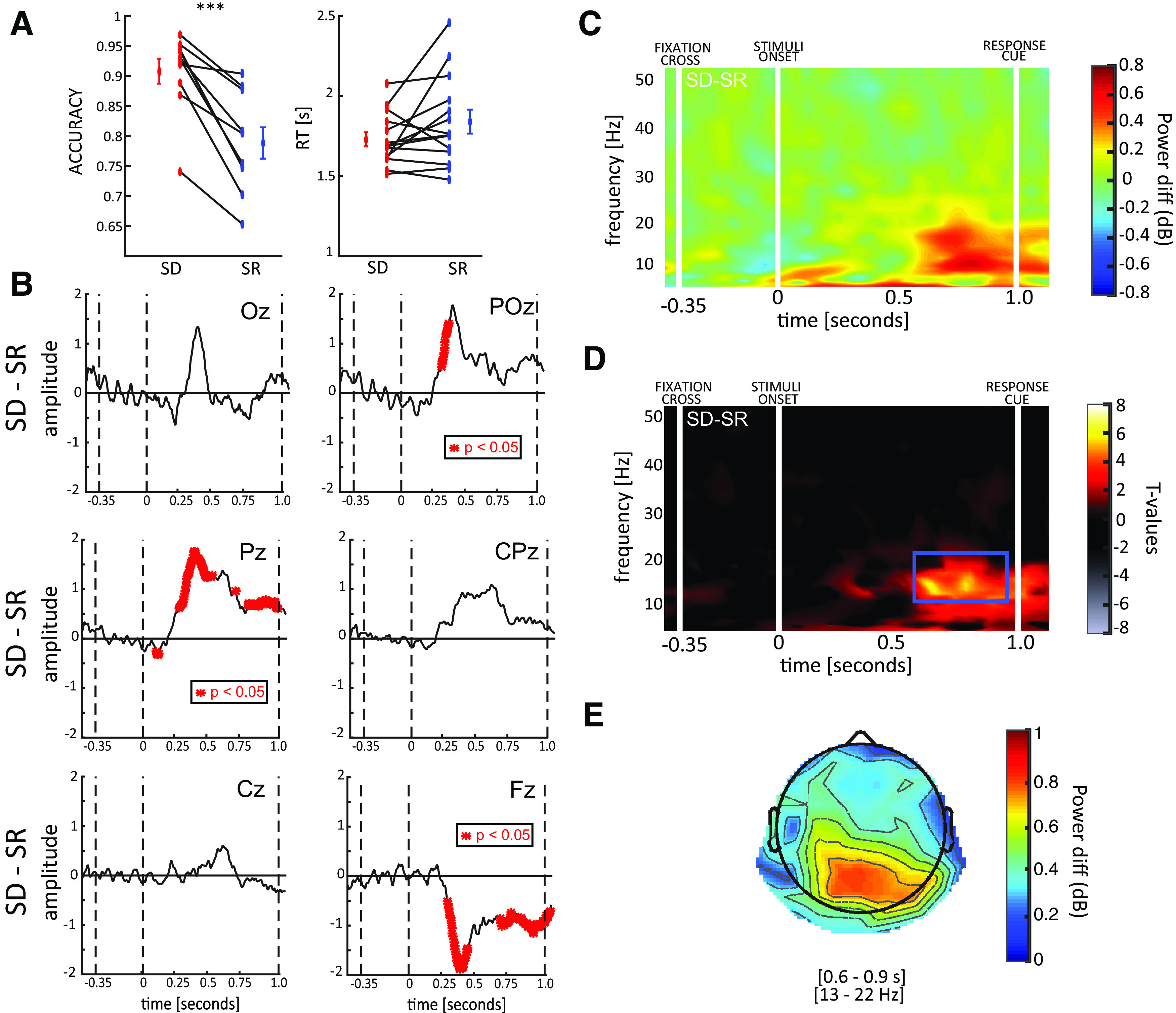
Pilot experiment results. ***A***, Behavioral results of the pilot experiment: left and right panel show accuracy and RTs for SD (red) and SR (blue) tasks. Differently than in the main task, in the pilot experiment participants performed significantly better in the SD than in the SR task (compare the accuracy between ***A*** and Fig. 2*B*). ***indicates Bayes Factor > 100. ***B***, Difference between SD and SR EPs. Red asterisk indicates time window significantly different from zero. ***C***, Difference between SD and SR power spectra: white lines indicate the stimulus onset and the response cue. ***D***, Testing the SD-SR difference against zero reveals a significant region in the low β band (13–21 Hz), before the response cue, in agreement with the results of the main experiment (Fig. 4). We reported a large effect size for this effect (one sample *t* test against zero averaging per each participant the values within the significant region, *t*_(13)_ = 7.049, *p* < 0.001, Cohen’s *d* = 1.820). ***E***, As in the main experiment, the SD-SR difference mostly involves occipital-parietal regions.

We performed a time-frequency analysis to try to identify differences between conditions observed in specific frequency bands commonly related to executive functions (e.g., visual working memory). For this purpose, we computed a baseline-corrected log-scale ratio between the two conditions (as shown in Fig. 4*A*), averaging over all electrodes. Remarkably, a point-by-point two-tailed *t* test corrected with cluster-based permutation test (Maris and Oostenveld, 2007) revealed a significantly larger activity in the low β band (16–24 Hz) in the SD condition between 250 and 950 ms after stimuli onset (Fig. 4*B*). We further quantify the magnitude of the effect by computing the effect size of a one sample *t* test against zero averaging per each participant the values within the significant region (*t*_(13)_ = 2.571, *p* = 0.023, Cohen’s *d* = 0.687). The topography of the effect spread mostly over parietal and occipital regions (Fig. 4*C*), mimicking the topography of the EPs analysis. As previously, these results confirm the prediction that the SD task may involve additional computational mechanisms beyond feedforward computations, possibly indexed by the β band oscillatory processes identified here. As previously, these results confirmed those from the pilot experiment (Fig. 5*D*,*E*), confirming the robustness of the effect also in the oscillatory domain. Below, we contextualize and substantiate our results in light of the relevant literature.

## Discussion

In this study, we confirmed in a series of two experiments a prediction from the computational study by Kim et al. (2018) that there exists an important dichotomy between visual reasoning tasks: While SR tasks can be solved by modern deep CNNs, SD tasks pose a significant challenge for these architectures, suggesting the need for additional computations beyond a feedforward cascade of filtering, rectification and normalization operations. Importantly, the result of these simulations does not allow us to formulate any prediction about the specific cortical processes involved in the two tasks. Nonetheless, it demonstrates a fundamental computational difference, which can be tracked in terms of its human brain neural correlates while subjects solve SD versus SR tasks (with difficulty objectively matched by an adaptive psychometric procedure). Remarkably, in both the pilot and the main experiment we found higher activity in the former task, in both EPs and oscillatory components. We interpret these differences as reflecting additional computations required by the SD task. We can speculate that these additional computations involve working memory and attention processes, which are lacking in feedforward architectures such as CNNs.

Additionally, it is possible to interpret our results in a broader context, by considering other tasks supposed to involve spatial attention, such as visual search. Previous experimental work suggested the need for re-entrant processes (Treisman and Gelade, 1980; Wolfe et al., 1989), and how increased activity in specific oscillatory components [i.e., low (22–34 Hz) and high (36–56 Hz) γ bands] are characteristic of these processes (Buschman and Miller, 2007; Phillips and Takeda, 2009). Accordingly, state-of-the-art computational models performing visual search and related tasks (e.g., instance segmentation) also employ attentional or recurrent mechanisms (Linsley et al., 2020), supporting the hypothesis that convolutional feedforward networks can benefit from recurrent mechanisms in solving visual reasoning tasks (Kreiman and Serre, 2020).

Computational evidence for the hypothesis that the SD task requires additional computational mechanisms beyond those needed to solve the SR task is provided by the results of the Siamese network simulations (Bromley et al., 1993). This feedforward network processes each stimulus item in a separate (CNN) channel and then passes the processed items to a single classifier network. Since each item is processed separately (the network is fed two images with only one item represented in each), this “oracle” architecture performs the task with item-segmentation processes automatically provided. Our results (as previously shown on another dataset by Kim and colleagues (Kim et al., 2018) demonstrate that such a feedforward network, once endowed with object individuation using the Siamese architecture, can easily learn to solve the SD task. In other words, this model simulates the beneficial effects of attentional selection, individuation and working memory by segregating the representations of each item. Our EEG results are compatible with this interpretation, with higher activity in the SD compared with the SR task, visible in both EPs and oscillatory frequency bands that have been previously related to attention and working memory (Nash and Fernandez, 1996; Benchenane et al., 2011; Lundqvist et al., 2018).

Previous work has shown that modulations of β band oscillations can be related to selective attention mechanisms (Buschman and Miller, 2007; Benchenane et al., 2011; Lee et al., 2013; Richter et al., 2018). Different attentional mechanisms may indeed be involved in the two tasks: the SR task could be solved by first grouping items and then determining the orientation of the group (Franconeri et al., 2012), whereas the SD task requires the individuation of the two items before comparison. In addition, our results are also consistent with differences in memory processes between the two tasks (de Fockert et al., 2001). One common assumption is that items that are grouped together (as in the SR task) occupy only one working memory slot (Franconeri et al., 2013; Clevenger and Hummel, 2014), whereas non-grouped items would each hold one slot, resulting in a larger working memory load. Previous literature showed that working memory can also be characterized by neuronal oscillatory signatures. Recent studies, for example, have demonstrated an interplay between β and γ band frequencies during working memory tasks (Lundqvist et al., 2016, 2018). Similarly, α and low β bands, not only increase with working memory load (Pesonen et al., 2007; Babiloni et al., 2004), but also in conjunction with the inhibition of competing visual memories in selective memory retrieval (Park et al., 2010; Waldhauser et al., 2012). Besides, previous studies have reported that increased oscillatory activity in the α band is a signature of attentional processes, and it can predict the likelihood of successful trials in many tasks (Händel et al., 2011; Klimesch, 2012; Nelli et al., 2017); however, in our current study we did not investigate differences between correct and incorrect trials, but between different types of tasks (involving SR or sameness judgment), after controlling for task difficulty . This could explain why α band amplitude differences were less prominent in our study. All considered, several lines of evidence point toward β oscillations as crucially involved in both attention and working memory related processes. These processes, therefore, might be part of the additional computational mechanisms required for SD tasks compared with SR tasks. Future work could more directly compare the attention and memory dependence of each task in human subjects.

That feedforward neural networks are limited in their ability to solve simple visual reasoning tasks is already being recognized by the computer vision and neuroscience communities (Yamins et al., 2013; Rajalingham et al., 2018; Kar et al., 2019). Current CNN extensions include modules for integrating local and global features (Chen et al., 2018) as well as recurrent neural architectures (Yang et al., 2018). Our results suggest that the human visual system also deploys additional computations beyond feedforward processes to successfully solve visual reasoning tasks. Rhythmic cortical oscillations in the β band represent the signatures of these additional computations, which may involve selective attention and working memory.

## Synthesis

Reviewing Editor: Ifat Levy, Yale University School of Medicine Decisions are customarily a result of the Reviewing Editor and the peer reviewers coming together and discussing their recommendations until a consensus is reached. When revisions are invited, a fact-based synthesis statement explaining their decision and outlining what is needed to prepare a revision will be listed below. The following reviewer(s) agreed to reveal their identity: Oren Shriki.

In this paper, the authors tested the hypothesis that the same-different (SD) task required more neural computations in the human visual system than the spatial relation (SR) task, even when task difficulty was equated. The authors compared the performance of human subjects and deep convolutional neural networks (dCNNs) on the two tasks, using stimuli that were statistically the same in both tasks. First, they replicated past results where deep dCNNs were better at performing the SR over the SD tasks. Also, achieving the SD task required the segmentation step during model simulation. Using EEG, the authors found higher P300 amplitude and higher beta power at the occipital electrodes in the SD compared to the SR tasks. They used these EEG data to support the idea that the SD task requires additional computational processes involving more attention and working memory, consistent with the modeling results.

The experimental work and computational modeling seem solid and the paper is clearly written. The overall framework of comparing deep networks to humans and examining differences in neural activity that may reflect different computational processes is appealing and interesting and could inspire future work. The authors also replicate their results using two sets of data, a commendable scientific practice, and one of the reviewers noted that the authors have done a good job responding to the reviewer’s comments in another journal.

At the same time, the existence of different EEG signatures for the two tasks considered is not very surprising and the conclusions drawn regarding the underlying neural mechanisms are indirect and very broad. It thus seems that the contribution of the work is more in the general framework and less in terms of novel insights into relevant neural processes, and this should be the focus of the paper.

In addition, the analysis of the neural networks focuses only on the learning process and the accuracy. To support the claims, it would be valuable to perform a more detailed analysis of the evolved networks using methods of explainability, such as Grad-CAM or LIME. These could highlight the important pixels in the decisions and illuminate if there is indeed a problem of ‘attention’ in the simple CNN compared to the Siamese one.

Minor comments:

1. The authors do a good job justifying the focus on P300 and beta power; however, it is not clear why early sensory ERPs and alpha power are not involved. Other studies have shown clear modulations of early sensory ERPs and changes in posterior alpha power to underlie better perceptual performance in visual tasks (Stoermer et al., 2009; Itthipuripat 2014; 2017; Nelli et al., 2017) as well as visuospatial attention and working memory processes (e.g., Van Voorhis and Hillyard, 1977; Woldorff et al., 1997; Itthipuripat, Sprague, Serences, 2019; Foster et al., 2016; 2017; reviewed in Klimesch 2012). Could it be that the additional processes required by SD compared to SR tasks are not simply ‘spatial’ attention or ‘spatial’ working memory, but some of those stimulus-specific and/or category-based domains?

2. It would be helpful to have diagrams of the different CNNs.

3. There has been substantial work on comparing CNNs with the visual system on different tasks such as invariant object recognition, e.g. from the lab of James DiCarlo at MIT, which could be cited in the Discussion.

4. If possible, it would be interesting to include in the Discussion similar predictions, based on CNNs, regarding EEG for other perceptual tasks, such as visual search or mental rotation.

## Author Response

Revision of Manuscript “Differential involvement of EEG oscillatory components in sameness vs. spatial-relation visual reasoning tasks” by Andrea Alamia, Canhuang Luo, Matthew Ricci, Junkyung Kim, Thomas Serre and Rufin VanRullen

We would like to thank the reviewers for their insightful comments which have helped improve the current manuscript. We are grateful for this opportunity to improve the manuscript. We hope the modifications will satisfy the reviewers and the editor. All the changes are highlighted in red in the revised manuscript. Please find below our detailed responses (in blue) to the reviewers.

Synthesis of Reviews:

Computational Neuroscience Model Code Accessibility Comments for Author (Required):

Please include a statement in the Materials and Methods section, under the heading “Code Accessibility", indicating whether and how the code can be accessed, including any accession numbers or restrictions, as well as the type of computer and operating system used to run the code.

Authors’ answer: We added a statement about the code accessibility. The code and data to perform the simulations are now available at this repository: https://github.com/artipago/SD-SR

Significance Statement Comments for Author (Required):

Please change the Author Summary into a Significance Statement, focusing on the importance and relevance of the research.

Authors’ answer: We modified the Author Summary into a Significance Statement in the revised manuscript, highlighting the interest and relevance of the results.

Comments on the Visual Abstract for Author (Required):

N/A

Synthesis Statement for Author (Required):

In this paper, the authors tested the hypothesis that the same-different (SD) task required more neural computations in the human visual system than the spatial relation (SR) task, even when task difficulty was equated. The authors compared the performance of human subjects and deep convolutional neural networks (dCNNs) on the two tasks, using stimuli that were statistically the same in both tasks. First, they replicated past results where deep dCNNs were better at performing the SR over the SD tasks. Also, achieving the SD task required the segmentation step during model simulation. Using EEG, the authors found higher P300 amplitude and higher beta power at the occipital electrodes in the SD compared to the SR tasks. They used these EEG data to support the idea that the SD task requires additional computational processes involving more attention and working memory, consistent with the modeling results.

The experimental work and computational modeling seem solid and the paper is clearly written. The overall framework of comparing deep networks to humans and examining differences in neural activity that may reflect different computational processes is appealing and interesting and could inspire future work. The authors also replicate their results using two sets of data, a commendable scientific practice, and one of the reviewers noted that the authors have done a good job responding to the reviewer’s comments in another journal.

At the same time, the existence of different EEG signatures for the two tasks considered is not very surprising and the conclusions drawn regarding the underlying neural mechanisms are indirect and very broad. It thus seems that the contribution of the work is more in the general framework and less in terms of novel insights into relevant neural processes, and this should be the focus of the paper.

Authors’ answer: We would like to thank the reviewers for their overall positive opinion on our work and for their suggestions. We believe the current version of the paper reflects this more general framework, as stated in the last sentence of the introduction, p.2-3 “We emphasize that the goal of the present study was not to identify the precise neural computations involved in the two tasks (which would naturally require a broader experimental set-up than a single EEG study), but rather to validate the hypothesis that SD involves additional computations relative to SR (even when the two tasks are equally difficult). We hope that this demonstration can be a first step towards characterizing the processes taking place in visual cortex during visual reasoning tasks, and designing more reliable and more human-like computational models", as well as in the discussion p.14 : “Importantly, the result of these simulations does not allow us to formulate any prediction about the specific cortical processes involved in the two tasks. Nonetheless, it demonstrates a fundamental computational difference, which can be tracked in terms of its human brain neural correlates while subjects solve SD vs. SR tasks (with difficulty objectively matched by an adaptive psychometric procedure”.

In addition, the analysis of the neural networks focuses only on the learning process and the accuracy. To support the claims, it would be valuable to perform a more detailed analysis of the evolved networks using methods of explainability, such as Grad-CAM or LIME. These could highlight the important pixels in the decisions and illuminate if there is indeed a problem of ‘attention’ in the simple CNN compared to the Siamese one.

Authors’ answer: We agree with the reviewers that methods such as Grad-CAM or LIME would help to shed light on the mechanisms involved in solving the two tasks, and we are grateful for the suggestion. We performed such analysis on the whole test-set for the simple CNNs trained on SD and SR tasks (as the Siamese network processes the two halves of the image independently, each sub-network would trivially highlight the only stimulus that it receives; therefore, we did not apply Grad-CAM to the Siamese network). Figure R1 below shows two random examples.

Figure R1 : left panels show the image with the 2 hexominoes fed to the networks, on the right panel the class activation map for the SR (above) and SD (below) tasks in case of positive classification using the GRAD-CAM method.

In order to quantify whether attention was distributed to both or one of the two stimuli, we computed the absolute normalized difference of the weighted sum of all the activations related to each hexominoes. Practically, if the network focuses on only one hexominoes (i.e. the activation is spread only around one part of the image), the difference will be close to 1, if the network split the activations equally between the two stimuli, the difference will be close to 0. As shown in figure R2, we were not able to find a clear difference between SD and SR tasks as a function of correct/wrong responses using this approach. This result may not be surprising, possibly due to the fact that in each image only a few pixels representing the hexominoes are non-zero, and all contribute to the final response. On the other hand, these results could indicate that the simple CNNs apply a similar strategy to the two tasks (SD and SR), a strategy that proves sufficient for the SR task but not the SD. In that sense, the presence of a similar pattern of attentional deployment remains compatible with our interpretation.

Figure R2: each panel shows the distribution of the differences for the SD (first row) and SR (second row) task when the networks perform correctly (left column) or not (right column; note that there were not enough wrong responses in the SR task to perform this analysis). The normalized difference represents a bias in processing one of the two stimuli, and it is considered as a proxy of the attentional focus for each network. Unfortunately, we were not able to observe any clear difference between SD and SR tasks, possibly due to the small number of active pixels in each image-for both tasks, it is likely that all non-zero pixels are being attended.

All in all we decided not to include this analysis in the current paper, with the aim of keeping the focus on the experimental results. However, we are grateful to the reviewers for their suggestions, as this interesting approach is likely to inspire future analyses in our follow-up studies (more focused on the machine learning side).

Minor comments:

1. The authors do a good job justifying the focus on P300 and beta power; however, it is not clear why early sensory ERPs and alpha power are not involved. Other studies have shown clear modulations of early sensory ERPs and changes in posterior alpha power to underlie better perceptual performance in visual tasks (Stoermer et al., 2009; Itthipuripat 2014; 2017; Nelli et al., 2017) as well as visuospatial attention and working memory processes (e.g., Van Voorhis and Hillyard, 1977; Woldorff et al., 1997; Itthipuripat, Sprague, Serences, 2019; Foster et al., 2016; 2017; reviewed in Klimesch 2012). Could it be that the additional processes required by SD compared to SR tasks are not simply ‘spatial’ attention or ‘spatial’ working memory, but some of those stimulus-specific and/or category-based domains?

Authors’ answer: We thank the reviewers for this interesting comment. Regarding the role of early sensory ERPs and alpha power, we point to the crucial difference between many of the aforementioned studies and our results. Specifically, it is true that in previous studies alpha oscillations as well as early ERPs have been shown to be correlated with task accuracy (“perceptual performance”), irrespective of the task at hand. The effect, however, is typically observed by contrasting correct with incorrect performance. On the other hand, in our work we did not focus on the difference between correct and incorrect trials, but we investigate the difference between two tasks (involving spatial relationship vs. identity judgments), equated for accuracy. We added a sentence in the discussion to highlight this important difference, see p.15 : “Besides, previous studies have reported that increased oscillatory activity in the alpha band is a signature of attentional processes, and it can predict the likelihood of successful trials in many tasks (Händel et al., 2011; Itthipuripat et al., 2017; Klimesch, 2012); however, in our current study we did not investigate differences between correct and incorrect trials, but between different types of tasks (involving spatial relationship or sameness judgment), after controlling for task difficulty . This could explain why alpha-band amplitude differences were less prominent in our study”.

Lastly, we exclude the possibility that the observed effect involves stimulus-specific and/or category-based domain because the stimuli we used in both tasks are exactly the same.

2. It would be helpful to have diagrams of the different CNNs.

Authors’ answer: We thank the reviewers for the useful suggestion. We added a panel in figure 1 including a diagram of the CNNs (see below). Figure R3 : New panel D of figure 1.

3. There has been substantial work on comparing CNNs with the visual system on different tasks such as invariant object recognition, e.g. from the lab of James DiCarlo at MIT, which could be cited in the Discussion.

Authors’ answer: We thanks the reviewer for the suggestion. We added references to some of the relevant work of James DiCarlo in the last paragraph of the discussion. P.15 : “That feedforward neural networks are limited in their ability to solve simple visual reasoning tasks is already being recognized by the computer vision and neuroscience communities (Kar et al., 2019; Rajalingham et al., 2017; Yamins et al., 2013)”.

4. If possible, it would be interesting to include in the Discussion similar predictions, based on CNNs, regarding EEG for other perceptual tasks, such as visual search or mental rotation.

Authors’ answer: We thanks the reviewer for the suggestion. We included in the first paragraph of the discussion a pointer to similar results in the visual search task - p.14: “

"Additionally, it is possible to interpret our results in a broader context, by considering other tasks supposed to involve spatial attention, such as visual search. Previous experimental work suggested the need for re-entrant processes (Treisman and Gelade, 1980; Wolfe et al., 1989), and how increased activity in specific oscillatory components (i.e. low [22-34Hz] and high [36-56Hz] gamma bands) are characteristic of these processes (Buschman and Miller, 2007; Phillips and Takeda, 2009). Accordingly, state-of-the-art computational models performing visual search and related tasks (e.g. instance segmentation) also employ attentional or recurrent mechanisms (Linsley et al., 2020), supporting the hypothesis that convolutional feedforward networks can benefit from recurrent mechanisms in solving visual reasoning tasks (Kreiman and Serre, 2020).”

References

Buschman TJ, Miller EK. 2007. Top-down versus bottom-up control of attention in the prefrontal and posterior parietal cortices. Science (80- ) 315:1860-1864. doi:10.1126/science.1138071

Händel BF, Haarmeier T, Jensen O. 2011. Alpha oscillations correlate with the successful inhibition of unattended stimuli. J Cogn Neurosci 23:2494-2502. doi:10.1162/jocn.2010.21557

Itthipuripat S, Cha K, Byers A, Serences JT. 2017. Two different mechanisms support selective attention at different phases of training. PLoS Biol 15. doi:10.1371/journal.pbio.2001724

Kar K, Kubilius J, Schmidt K, Issa EB, DiCarlo JJ. 2019. Evidence that recurrent circuits are critical to the ventral stream’s execution of core object recognition behavior. Nat Neurosci 22:974-983. doi:10.1038/s41593-019-0392-5

Klimesch W. 2012. Alpha-band oscillations, attention, and controlled access to stored information. Trends Cogn Sci. doi:10.1016/j.tics.2012.10.007

Kreiman G, Serre T. 2020. Beyond the feedforward sweep: feedback computations in the visual cortex. Ann N Y Acad Sci. doi:10.1111/nyas.14320

Linsley D, Ashok AK, Govindarajan LN, Liu R, Serre T. 2020. Stable and expressive recurrent vision models.

Phillips S, Takeda Y. 2009. Greater frontal-parietal synchrony at low gamma-band frequencies for inefficient than efficient visual search in human EEG. Int J Psychophysiol 73:350-354. doi:10.1016/j.ijpsycho.2009.05.011

Rajalingham R, Issa EB, Schmidt K, Kar K, DiCarlo JJ. 2017. Feedforward deep neural networks diverge from humans and monkeys on core visual object recognition behaviorAnnual Conference on Cognitive Computational Neuroscience. pp. 1-2.

Treisman A, Gelade G. 1980. A feature integration theory of attention. Cogn Psychol 12:97-136.

Wolfe JM, Cave KR, Franzel SL. 1989. Guided Search: An Alternative to the Feature Integration Model for Visual Search. J Exp Psychol Hum Percept Perform 15:419-433. doi:10.1037/0096-1523.15.3.419

Yamins D, Hong H, Cadieu C, Dicarlo JJ. 2013. Hierarchical modular optimization of convolutional networks achieves representations similar to Macaque IT and human ventral streamAdvances in Neural Information Processing Systems.
